# The therapeutic effects of human embryonic stem cells-derived immunity-and-matrix regulatory cells on membranous nephropathy

**DOI:** 10.1186/s13287-022-02917-w

**Published:** 2022-06-07

**Authors:** Hui-song Zhou, Zhao Cui, Hui Wang, Ting-ting Gao, Liu Wang, Jun Wu, Zu-ying Xiong, Jie Hao, Ming-hui Zhao

**Affiliations:** 1 Renal Division, Peking University First Hospital; Institute of Nephrology, Peking University; Key Laboratory of Renal Disease, Ministry of Health of China; Key Laboratory of CKD Prevention and Treatment, Ministry of Education of China, Beijing, 100034 China; 2 Renal Division, Peking University Shenzhen Hospital, Shenzhen, 518000 China; 3 Department of Electron Microscopy, Peking University First Hospital, Beijing, 100034 China; 4 National Stem Cell Resource Center, Institute of Zoology, Chinese Academy of Sciences, Beijing, 100101 China; 5 State Key Laboratory of Stem Cell and Reproductive Biology, Institute of Zoology, Chinese Academy of Sciences, Beijing, 100101 China; 6 Beijing Institute for Stem Cell and Regenerative Medicine, Beijing, 100101 China; 7 Institute for Stem Cell and Regeneration, Chinese Academy of Sciences, Beijing, 100101 China; 8 University of Chinese Academy of Sciences, Beijing, 100101 China; 9 Peking-Tsinghua Center for Life Sciences, Beijing, 100080 China

**Keywords:** Membranous nephropathy, Immunity-and-matrix regulatory cells, Treatment, Regulatory T cells, IL-10

## Abstract

**Background:**

Primary membranous nephropathy (MN) is a kidney-specific autoimmune disease. Human embryonic stem cells-derived immunity-and-matrix regulatory cells (hESC-IMRCs) have immunoregulatory functions. We hypothesized that hESC-IMRCs might have therapeutic effects on MN and be a potential treatment in clinical practice.

**Methods:**

Rats of Heymann nephritis were injected with sheep anti-rat Fx1A serum. hESC-IMRCs were intravenously administrated upon the detection of proteinuria, with 6 × 10^6^ cells (high-dose) or 3 × 10^6^ cells (low-dose) in 1 ml every other day. Splenocytes and IMRCs were co-cultured at different times and ratios. Cell types and cytokines were detected by flow cytometry and enzyme-linked immunosorbent assay.

**Results:**

The urinary protein of rats with Heymann nephritis was reduced remarkably to a level comparable to negative controls, in both low-dose (45.6 vs. 282.3 mg/d, *P* < 0.001) and high-dose (35.2 vs. 282.3 mg/d, *P* < 0.001) hESC-IMRC treatment groups. IgG and C3 deposit, glomerular basement membrane thickness and foot process effacement were alleviated and the reduced podocin was recovered in the kidneys. The proportions of CD4 + CD25 + T cells in circulation and spleen were increased, and the circulating level of IL-10 was increased, after IMRC interventions. IL-17 and TNF-α were reduced after IMRCs treatments. IL-10 increased remarkably in the co-culture supernatant of lymphocytes and IMRCs at 48 h with ratio 10:1.

**Conclusions:**

The intravenously delivered hESC-IMRCs alleviated proteinuria and kidney injuries of Heymann nephritis, by their immunosuppressive functions through regulatory T cells and IL-10. These pre-clinical results indicate that IMRCs worth careful consideration for human trials in the treatment of MN.

**Supplementary Information:**

The online version contains supplementary material available at 10.1186/s13287-022-02917-w.

## Introduction

Primary membranous nephropathy (MN) is an autoimmune disease and a common cause of nephrotic syndrome in adults. It is characterized by the subepithelial formation of immune deposits containing antigens, IgG, and complement components [1, 2]. The annual incidence of primary MN has been estimated as 1.2 per 100,000 adults in the United States and European countries [3]; in contrast, a significant increase of patients with primary MN was witnessed in mainland China [4]. The main treatments of MN include supportive therapies using angiotensin-converting enzyme inhibitors or angiotensin II receptor blockers for lowering blood pressure and reducing urinary protein, and immunosuppressive therapies with or without steroids [5, 6]. Even having received these treatments, some patients present with continuous nephrotic syndrome and kidney dysfunction [5, 6].

Stem cells are unspecialized cells with self-renewal and differentiation potentials. The therapy using stem cells is emerging to cure various inflammatory, degenerative and autoimmune diseases [7–10]. Mesenchymal stem cells (MSCs) present with an immune regulatory role in both adaptive and innate immune systems and have been widely used in clinical trials as immunosuppressive agents for autoimmune and inflammatory diseases, including graft-versus-host disease, multiple sclerosis, systemic lupus erythematosus, crescentic glomerulonephritis, acute kidney injury and chronic kidney disease [11–17]. Human embryonic stem cells (hESCs)-derived MSC-like cell population, which is called immunity and matrix-regulatory cells (IMRCs) [18], has unique abilities in modulating immunity and regulating extracellular matrix production, compared to regular MSC populations. On the one hand, IMRCs resemble MSCs in their self-renewal and tri-lineage differentiation capacity. On the other hand, compared to primary umbilical cord mesenchymal stem cells, IMRCs display a higher consistency in quality and more potent immunomodulatory and anti-fibrotic functions.

In the present study, we administrated IMRCs treatments on Heymann nephritis, with the aim to explore the therapeutic effects and immune regulatory mechanism of MSCs on MN.

## Materials and methods

### Generation of hESC-IMRCs

The hESC-IMRCs were generated by National Stem Cell Resource Center, Institute of Zoology, Chinese Academy of Sciences, Beijing, as described previously [18]. In brief, clinical hESCs were maintained in Essential 8™ basal medium (E8) on vitronectin-coated plates before dissociation into small clumps to form human embryoid bodies (hEBs) for 5 days. Subsequently, hEBs were transferred onto vitronectin-coated plates and cultured for 14 additional days. The hEBs outgrowth cells were dissociated and passaged continuously in IMRCs Medium. After 5 passages, IMRCs were harvested for characterization. IMRCs possessed fibroblast-like morphology and maintained diploid karyotypes at passage 5. Copy-number variation analysis by whole-genome sequencing showed no chromosomal aneuploidies, large deletions nor duplication fragments. IMRCs showed an expression profile greatly differed from hESCs, and closely resembled primary umbilical cord-derived MSCs (UCMSCs). All pluripotency genes, mesendoderm genes, and ectoderm genes were extinguished. IMRCs expressed MSC-specific genes and surface markers, while negative of hematopoietic surface markers. IMRCs displayed the ability to undergo tri-lineage differentiation into mesenchymal tissues, such as adipocytes, chondroblasts and osteoblasts. The proliferation rate of IMRCs was higher than that of UCMSCs at passage 15. IMRCs were generally smaller than UCMSCs.

### Induction of passive Heymann nephritis

Sprague–Dawley (SD) rats, male, with body weights of 100–120 g, were immunized using sheep anti-rat Fx1A serum (Probetex, Inc., Texas, USA) 0.7 ml/100 g by a single intravenous injection. Twenty-four-hour urine and blood samples were collected before and after immunization at each week. All rats were killed at the end of week 7 after immunization according to our previous experience. Kidney tissues, spleens and blood samples were collected at sacrifice.

### Treatment with hESC-IMRCs

Treatment was administrated upon the detection of proteinuria by urine dipsticks (Urit, Guilin, China) (approximately 3 weeks after immunization). The rats were randomly divided into three groups with the level of proteinuria comparable. For the high-dose treatment group (*n* = 10), hESC-IMRCs were injected into rats intravenously at 6 × 10^6^ cells in 1 ml every other day from week 3 to week 6 after immunization. For low-dose treatment group (*n* = 10), hESC-IMRCs were administrated at 3 × 10^6^ cells in 1 ml with the same method. Sterile physiological saline treatment was used on rats of disease controls (*n* = 10).

### Evaluation of disease severity

The levels of urinary protein, serum creatinine, blood urea nitrogen, cholesterol, and triglyceride were analyzed by an automatic biochemical analyzer (UniCel DxC 600 Synchron; Beckman Coulter, Inc).

Kidney tissues were examined by light microscopy, immunofluorescence, and electron microscopy. For light microscopy, kidney tissues were fixed in 10% buffered formalin and embedded in paraffin. Kidney sections (3 μm) were stained with hematoxylin and eosin, periodic acid-Schiff, periodic acid-sliver methenamine and Masson’s trichrome. At least 60 glomeruli in each tissue were assessed. For immunohistochemical staining, anti-nephrin antibodies (1:1000, Abcam, Cambridge, UK) were incubated overnight at 4 °C. Secondary antibodies were incubated at room temperature for 1 h. DAB color was developed and observed. Eight non-overlapping fields of view were randomly selected under high magnification (× 400). Average Optical Density Value = Integrated Optical Density (IOD)/Positive Area Distribution (AREA) (Image Pro Plus, Media Cybernetics, Rockville, MD, USA).

For immunofluorescence, cryosections (5 μm) were fixed in acetone, blocked with 3% bovine serum albumin in PBS, and stained with fluorescein isothiocyanate (FITC)-conjugated goat anti-rat IgG (1:100, Jackson ImmunoResearch Laboratories Inc., Seattle, WA, USA) or FITC-conjugated goat anti-rat C3c (1:50, Abcam, Cambridge, UK).

For electron microscopy, kidney tissues were immersed in cold 5% glutaraldehyde, postfixed in osmium tetroxide, and embedded in Epon 812. Sections were stained with uranyl acetate and lead citrate and examined on an electron microscope (JEM-1230; Jeol, Tokyo, Japan). For evaluation of foot process width per length of GBM, two glomeruli were randomly selected from each kidney sample. A total of 20–25 representative non-overlapping digital micrographs were taken from each glomerulus under × 10,000 magnification. The number of foot processes per micrometer of GBM and GBM thickness was calculated using Photoshop or ImageJ. The arithmetic mean of foot process width was calculated as: (*π*/4) × (ΣGBM length/Σnumber of foot process) [19], where ΣGBM length was the total GBM length measured in one glomerulus and Σnumber of foot process was the total number of one glomerulus foot process counted. The correction factor (π/4) had been applied to correct the random orientation when the foot processes were sectioned.

### Labeled hESC-IMRCs distribution in rats

hESC-IMRCs were labeled with GFP (Abcam, Cambridge, UK). The rats of passive Heymann nephritis were killed at 1, 12, 24, and 48 h after injection of GFP-hESC-IMRCS at 3 × 10^6^ cells in 1 ml. The negative control rats were injected with sterile physiological saline used as blank control. Major organs (heart, liver, spleen, lung, kidneys, intestines) were exposed to a Bruker FX PRO imaging system (Bruker Corporation, Billerica, USA) with excitation at 465 nm and emission at 520 nm. For analysis, ROIs were drawn around the organs and the average fluorescence intensity was measured in each ROI using Living image 4.0 software (Caliper Life Sciences, Hopkinton, USA). The normalized fluorescence intensity (NFI) was calculated and compared at different time points.

$${\text{NFI}} = \frac{{\text{Organ fluorescence intensity - background fluorescence intensity}}}{{\text{Blank fluorescence intensity}}}$$

### Flow cytometric analysis

The spleens were aseptically removed and disrupted by mechanical dissociation. After filtration through a 100-μm nylon screen, splenocytes were collected, washed, and suspended with red blood cell lysing buffer. The splenocytes were suspended in diluted staining perm wash buffer (BioLegend, San Diego, CA) at a concentration of 1 × 10^7^ cells/ml. Cell suspensions or blood samples 100 μl were stained with FITC-anti-CD3 (1:500, BioLegend, San Diego, CA, USA), APC-anti-CD4 (1:100, BioLegend), PerCP-anti-CD8a (1:100, BioLegend), PE-anti-CD25 (1:100, BioLegend) and APC-Cy7 anti-CD45 (1:100, BioLegend) or isotype-matched controls (1:200, BioLegend), according to the manufacturer’s instructions. The samples were mixed gently, incubated for 20 min at 4 °C in the darkness and subsequently analyzed using FACS Verse flow (Becton Dickinson, CA, USA). The gating strategies are shown in Additional file 1.

### Co-culture and measurement of cytokine production

The splenocytes were centrifuged at 1500 rpm for 3 min and resuspended with IMRC culture medium (Chinese Academy of Sciences) at a density of 1 × 10^6^ cells/ml. Splenocytes cells and hESC-IMRCs were co-cultured at a ratio of 10:1 in 96-well culture plates. The cell concentration of hESC-IMRCs was ranged from 2 × 10^3^ cells to 2 × 10^5^ cells. The culture supernatants were collected at 24 h, 48 h and 72 h and stored at − 80 °C until assayed for IFN-γ, IL-2 and IL-10, by commercial enzyme-linked immunosorbent assay kits (ELISA, Biotech Co., Beijing, China), according to the manufacturer’s instructions.

The plasma levels of IL-17A, TNF-α, IL-10, IL-2, IL-4, IL-6 and complement C3 were measured by commercial ELISA kits (Abcam, Cambridge, UK), according to the manufacturer's protocols.

### Statistical analyses

SPSS version 22.0 (SPSS Inc., Chicago, IL, USA) was used for statistical analysis. Results were expressed as mean ± SD. Differences in quantitative parameters were assessed using separate individual *t* test for data that were normally distributed or Mann–Whitney *U* test for data that were not normally distributed. *P* values < 0.05 were considered statistically significant.

### Study approval

All animal experiments were approved by the Experimental Animal Ethics Committee of Peking University First Hospital, Beijing, China.

## Results

### Effect of hESC-IMRCs on proteinuria of Heymann nephritis

**Fig. 1:**
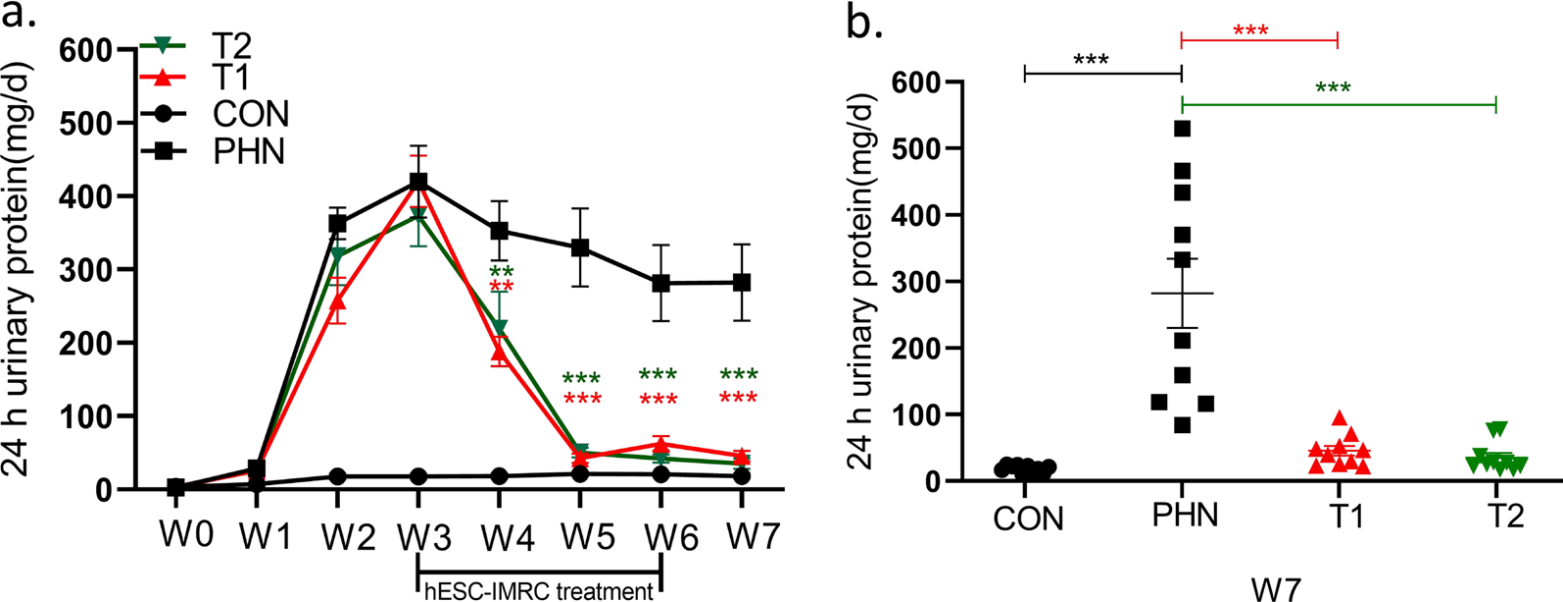
The clinical parameters of rats with passive Heymann nephritis and those after hESC-IMRC treatments. The rats developed heavy proteinuria from week 3 after the passive immunization of anti-rat Fx1A serum (**a**). Then IMRCs were intravenously administrated every other day with 3 × 10^6^ cells (T1, *n* = 10) or 6 × 10^6^ cells (T2, *n* = 10) in 1 ml from week 3 to week 6. The urinary protein was significantly reduced in both groups, compared to that in disease controls (*) with sterile physiological saline treatment (PHN, *n* = 10), and was similar like that in negative controls (CON, *n* = 10) at week 7 (**b**). *P < 0.05; **P < 0.01; ***P < 0.001

The rats developed heavy proteinuria in the third week after the passive immunization of anti-rat Fx1A serum, with urinary protein much higher than negative controls (404.4 ± 23.8 vs. 17.6 ± 2.4 mg/d, *P* < 0.001, Fig. 1). Then IMRC treatment was administrated upon the detection of proteinuria, approximately 3 weeks after immunization.

In the high-dose treatment group (*n* = 10), hESC-IMRCs were intravenously administrated every other day with 6 × 10^6^ cells in 1 ml from week 3 to week 6. After treatment, urinary protein was significantly reduced at week 4 (219.1 ± 50.6 vs. 352.5 ± 40.5 mg/d, *P* = 0.049), week 5 (50.3 ± 6.5 vs. 330.0 ± 53.3 mg/d, *P* = 0.001), week 6 (42.1 ± 5.5 vs. 281.5 ± 51.8 mg/d, *P* < 0.001), and week 7 (35.2 ± 7.2 vs. 282.3 ± 51.8 mg/d, *P* < 0.001), compared to those in disease control group with sterile physiological saline treatment (*n* = 10) (Fig. 1).

In the low-dose treatment group (*n* = 10), hESC-IMRCs were administrated at 3 × 10^6^ cells in 1 ml with the same method. After treatment, urinary protein was significantly reduced at week 4 (188.3 ± 20.1 vs. 352.5 ± 40.5 mg/d, *P* = 0.008), week 5 (42.5 ± 6.2 vs. 330.0 ± 53.3 mg/d, *P* < 0.001), week 6 (62.4 ± 10.1 vs. 281.5 ± 51.8 mg/d, *P* = 0.001), and week 7 (45.6 ± 7.4 vs. 282.3 ± 51.8 mg/d, *P* < 0.001), compared to those in disease controls (Fig. 1). There was no difference in proteinuria between the two groups of different IMRC doses (*P* > 0.05).

### Effect of hESC-IMRCs on kidney pathologic injuries of Heymann nephritis

**Fig. 2:**
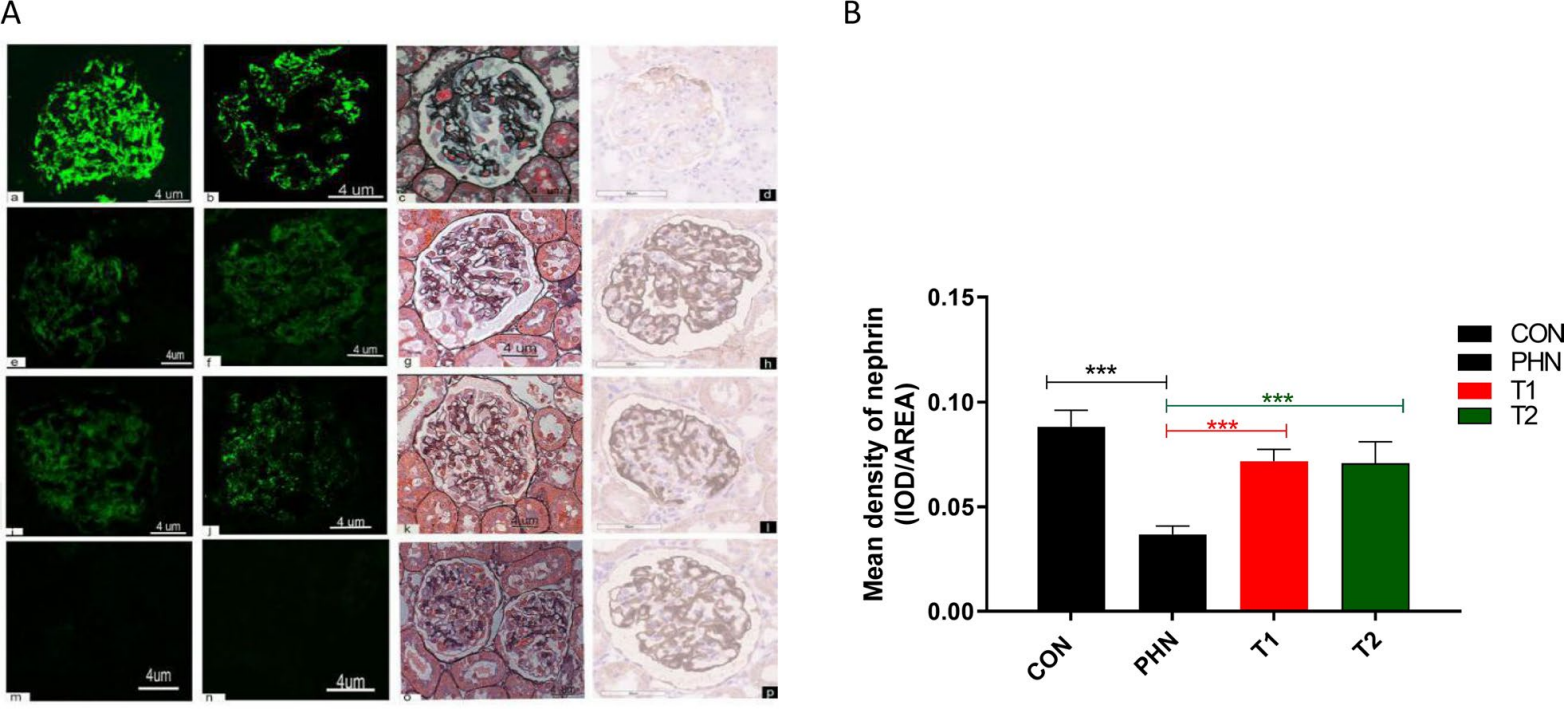
The kidney pathologic injuries of rats with passive Heymann nephritis and those after hESC-IMRC treatments. The kidneys of rats with passive Heymann nephritis showed diffusive granular staining of IgG (a) and C3 (b) along the glomerular capillary walls (× 400), glomerular basement membrane thickening and scattered spike formation (c), and decreased level of nephrin (d) (**A**, × 400). After hESC-IMRC treatments, the deposits of IgG (e, i) and C3 (f, j), the thickening and spikes (g, k) of basement membrane were alleviated, and the levels of nephrin were increased (h, l), in both the high-dose IMRC group (6 × 10^6^ cells) and the low-dose IMRC group (3 × 10^6^ cells). No obvious kidney injury was observed in negative controls (m–p). Nephrin expression in kidneys was reduced in rats of Heymann nephritis (**B**), which was recovered in both the low-dose (3 × 10^6^ cells, T1, *n* = 8) and high-dose (6 × 10^6^ cells, T2, *n* = 8) groups of IMRC treatments. *P < 0.05; **P < 0.01; ***P < 0.001

In the disease control group, diffuse granular staining of IgG and C3 was shown along the glomerular capillary walls. Their intensities were weaker in both high-dose and low-dose IMRC treatment groups at week 7 (Fig. 2). The level of nephrin was decreased in the disease controls (AOD: 0.0369 ± 0.0039 vs. 0.0884 ± 0.0078, *P* < 0.001) compared to the negative control group. After hESC-IMRC treatment, it was significantly increased in both the high-dose group (0.0719 ± 0.0055 vs. 0.0369 ± 0.0039, *P* < 0.001) and the low-dose group (0.0709 ± 0.0102 vs. 0.0369 ± 0.0039, *P* < 0.001) (Fig. 2).

**Fig. 3:**
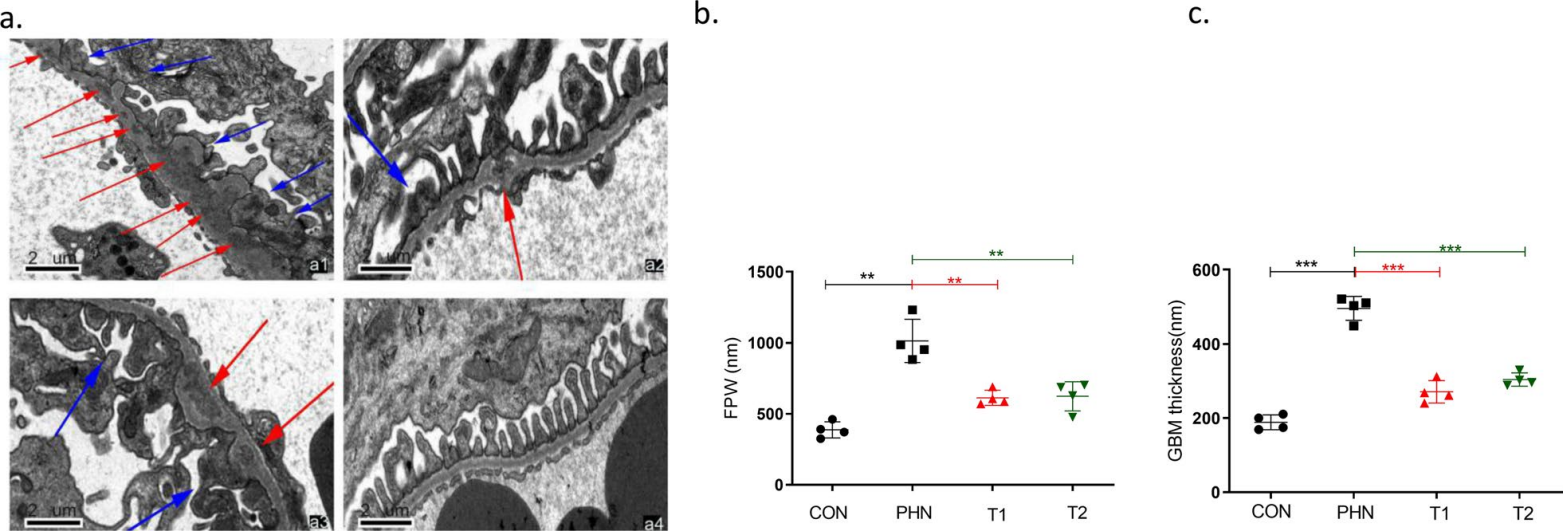
Analysis of glomerular basement membrane. Electron-dense deposits in the subepithelial area (red asterisks) and the podocyte foot processes diffusive fusion (blue asterisks) on electron microscopy (**a1**, × 10,000). The electron-dense deposits and the podocyte foot processes fusion (**a2**, **a3**) were alleviated, in both the high-dose IMRC group (6 × 10^6^ cells) and the low-dose IMRC group (3 × 10^6^ cells). No obvious kidney injury was observed in negative controls (**a4**). Quantitative analysis of podocyte foot process width (FPW) and glomerular basement membrane thickness. The foot process width (**b**) in low-dose IMRC group (3 × 10^6^ cells, T1, *n* = 4) and that in high-dose IMRC group (6 × 10^6^ cells, T2, *n* = 4) was significantly decreased at week 7, and was comparable to that in negative controls (*n* = 4). The thickness of glomerular basement membrane (**c**) in high-dose IMRC group and low-dose IMRC group was significantly decreased at week 7, and was comparable to that in negative controls. *P < 0.05; **P < 0.01; ***P < 0.001

In the disease control group, electron microscopy showed diffusive and extensive podocyte foot processes effacement, electron-dense deposits under epithelial cells and glomerular basement membranes diffuse thickening. These kidney injuries were alleviated in IMRC treatment groups of both doses (Fig. 3). Quantitative analysis of foot process width showed that compared to disease controls, the foot process width in high-dose IMRC group (624.0 ± 103.1 vs. 1014.3 ± 152.6 nm, *P* = 0.005) and that in low-dose IMRC group (613.6 ± 53.1 vs. 1014.3 ± 152.6 nm, *P* = 0.003) were significantly decreased at week 7 (Fig. 3). Quantitative analysis of glomerular basement membrane thickness showed that compared to the disease controls, the basement membrane thickness in high-dose IMRC group (304.4 ± 18.0 vs. 496.4 ± 32.2 nm, *P* < 0.001) and that in low-dose IMRC group (271.0 ± 30.0 vs. 496.4 ± 32.2 nm, *P* < 0.001) was significantly decreased at week 7 (Fig. 3).

### *The immune regulation effects of hESC-IMRCs *in vivo

We assessed the level of circulating IL-17A, TNF-α, IL-10, IL-2, IL-4, and IL-6 among the four groups of rats.

**Fig. 4:**
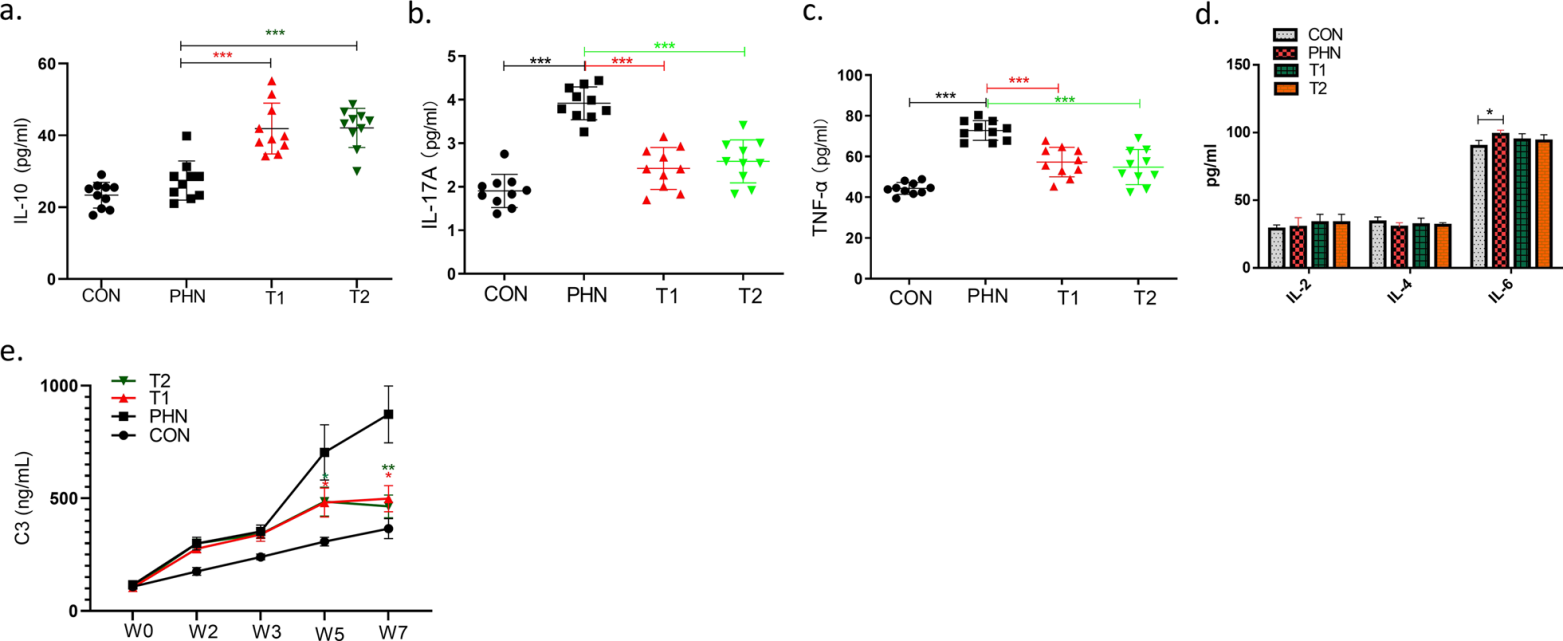
The plasma level of cytokines and C3 in Heymann nephritis rats and those after hESC-IMRC treatments. The plasma level of IL-10 (**a**) was increased in both groups of lower-dose (T1) and high-dose (T2) of IMRC treatments. The plasma level of IL-17A, TNF-α, and C3 was decreased in both groups (**b**, **c**, **e**). The plasma level of IL-2, IL-4, IL-6 was comparable among the four groups (**d**). *P < 0.05; **P < 0.01; ***P < 0.001

In the disease control group, circulating IL-17A was significantly increased, compared to that of negative controls at week 7 (3.92 ± 0.38 vs. 1.91 ± 0.38 pg/ml, *P* < 0.001) (Fig. 4). It was decreased in both high-dose IMRC group (2.58 ± 0.49 vs. 3.92 ± 0.38 pg/ml, *P* < 0.001) and low-dose IMRC group (2.42 ± 0.48 vs. 3.92 ± 0.38 pg/ml, *P* < 0.001), compared to disease controls at week 7.

The circulating TNF-α was significantly increased in the disease control group, compared to that of negative controls at week 7 (73.1 ± 5.4 vs. 44.4 ± 3.2 pg/ml, *P* < 0.001) (Fig. 4). It was decreased in both high-dose IMRC group (57.9 ± 9.2 vs. 73.1 ± 5.4 pg/ml, *P* < 0.001) and low-dose IMRC group (59.2 ± 7.3 vs. 73.1 ± 5.4 pg/ml, *P* < 0.001), compared to disease controls at week 7.

IL-10 was comparable between the disease and negative control groups (27.4 ± 5.4 vs. 23.4 ± 3.6 pg/ml, *P* = 0.063) (Fig. 4). However, it was increased in both high-dose IMRC group (41.9 ± 7.1 vs. 27.4 ± 5.4 pg/ml, *P* < 0.001) and low-dose IMRC group (42.1 ± 5.5 vs. 27.4 ± 5.4 pg/ml, *P* < 0.001), compared to disease controls at week 7.

The plasma level of IL-2, IL-4, IL-6 were comparable among the four groups (Fig. 4).

The plasma level of C3 was significantly increased in disease controls. After IMRC treatments, it was decreased in both high-dose group (498.6 ± 157.9 vs. 872.7 ± 126.5 ng/ml, *P* = 0.016) and low-dose group (464.2 ± 50.0 vs. 872.7 ± 126.5 ng/ml, *P* = 0.010), compared to that of disease controls at week 7. There was no difference in plasma level of C3 between the two groups of different IMRC doses (*P* > 0.05) (Fig. 4).

**Fig. 5:**
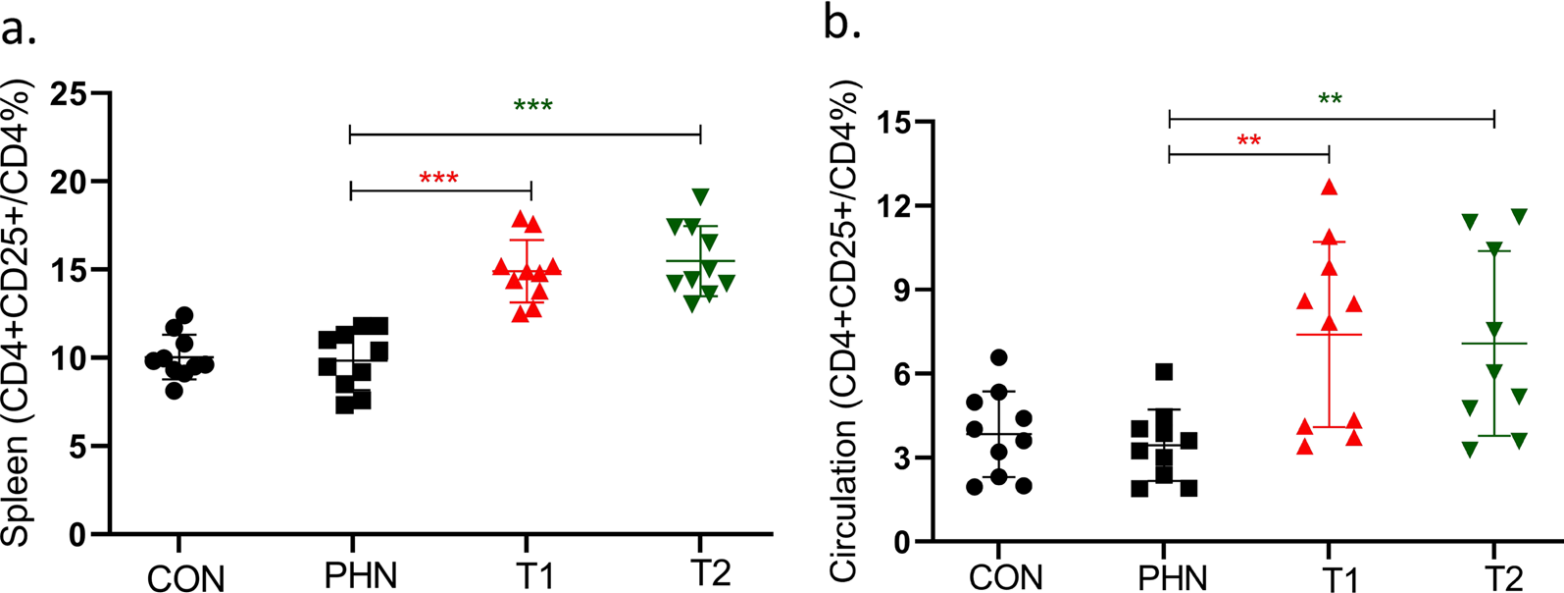
The proportion of CD4 + CD25 + T cells in circulation and in spleen. The proportion of CD4 + CD25 + T cells in CD4 + T lymphocyte cells was higher in circulation of the two IMRC treated rat groups (**a**). It was also higher in spleen of the two IMRC treated rat groups (**b**). *P < 0.05; **P < 0.01; ***P < 0.001

The proportion of CD4^+^CD25^+^ T cells in CD4^+^ T lymphocyte cells was higher in circulation of the two IMRC-treated groups (high-dose: 7.4 ± 3.3 vs. 3.4 ± 1.3%, *P* = 0.002; low-dose: 7.2 ± 3.1 vs. 3.4 ± 1.3%, *P* = 0.002), compared to that of disease control group. The proportion of CD4^+^CD25^+^ T cells was also higher in spleen of the two IMRC-treated groups (high-dose: 15.5 ± 2.0 vs. 10.0 ± 1.3%, *P* < 0.001; low-dose: 14.9 ± 1.8 vs. 10.0 ± 1.3%, *P* < 0.001), compared to that of disease control group (Fig. 5).

### hESC-IMRCs engrafted in rats target tissues

**Fig. 6:**
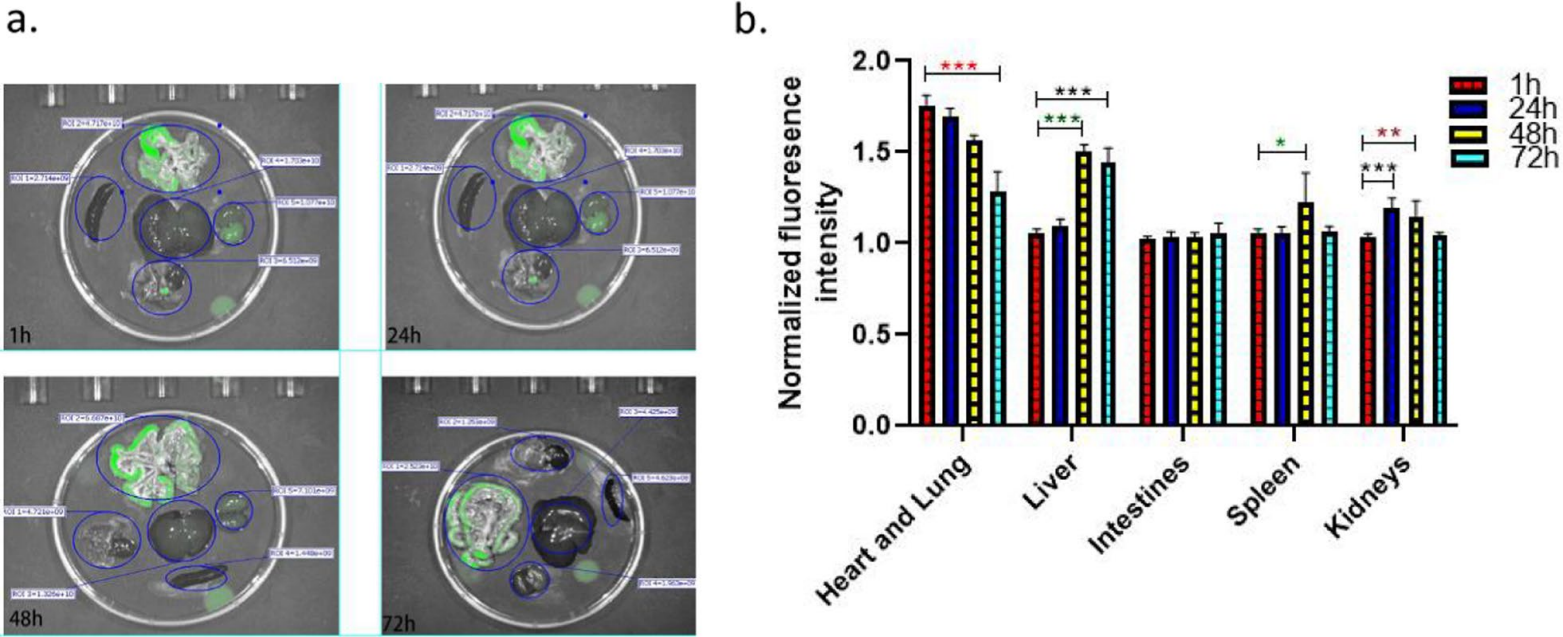
hESC-IMRCs engrafted in PHN rats target tissues. Ex vivo fluorescence imaging (**a**) and corresponding fluorescence intensities of major organs collected from PHN rats injected with hESC-IMRCs at different time intervals (**b** Heart and lung, liver, intestines, spleen, kidneys, respectively)

To assess the hESC-IMRCs engrafted in PHN rats target tissues, an ex vivo tissue-specific localization study was performed by fluorescence imaging of major organs at 1 h, 24 h, 48 h and 72 h post injection of GFP-hESC-IMRCs (Fig. 6).

The results showed that hESC-IMRCs were rapidly accumulated in heart and lungs at 1 h (1.8 ± 0.06) after administration and sustained for at least 72 h (1.3 ± 0.1), much higher than those in the other organs (liver, spleen, intestines, kidneys, *P* < 0.001) at the corresponding time. The fluorescence in kidneys was increased gradually at 24 h (1.2 ± 0.05, *P* < 0.001) and 48 h (1.1 ± 0.08, *P* = 0.016), compared to that at 1 h (1.0 ± 0.02). The fluorescence in liver (1.5 ± 0.04) and spleen (1.2 ± 0.2) was increased at 48 h.

### *The immune regulation effect of hESC-IMRCs *in vitro

**Fig. 7:**
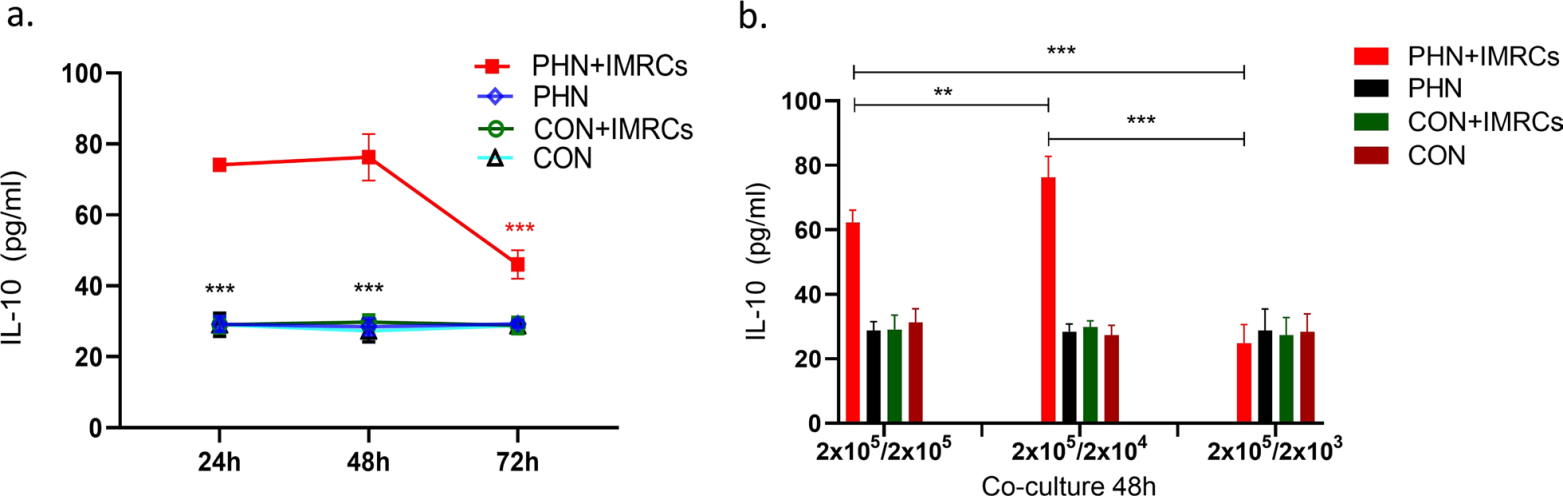
IL-10 production in the supernatant of splenic lymphocytes cocultured with hESC-IMRCs or not. IL-10 was increased after 24 h, 48 h, and 72 h of co-culture of splenic lymphocytes from disease rats with hECS-MSCs at a density ratio of 10:1 (**a**). IL-10 was higher in the co-culture supernatant with the ratio of splenocytes to IMRCs as 10:1, than that in the co-culture of splenocytes to lMRCs ratios as 1:1 and 100:1. *P < 0.05; **P < 0.01; ***P < 0.001

hESC-IMRCs were co-cultured with the splenic lymphocytes from Heymann nephritis rats at a density ratio of 10:1 (lymphocytes: MSCs) (Fig. 7). IL-10 expression in the supernatant was analyzed at different time points. After 24 h of co-culture, it was 74.1 ± 1.3 pg/ml in the co-culture supernatant, much higher than that in the supernatant of splenic lymphocytes from disease rats without IMRC co-culture (29.2 ± 2.1 pg/ml, *P* = 0.004), splenic lymphocytes from negative control rats with IMRC co-culture (29.0 ± 2.2 pg/ml, *P* = 0.004), and splenic lymphocytes from negative control rats without IMRC co-culture (29.2 ± 3.3 pg/ml, *P* = 0.004). After 48 h, the level of IL-10 was 76.3 ± 6.5 pg/ml in the supernatant of splenic lymphocytes from disease rats with IMRC co-culture, which was significantly higher than that of splenic lymphocytes from disease rats without IMRC co-culture (28.5 ± 2.3 pg/ml, *P* = 0.004), splenic lymphocytes from negative control rats with IMRC co-culture (29.8 ± 2.0 pg/ml, *P* = 0.004), and splenic lymphocytes from negative control rats without IMRC co-culture (27.3 ± 3.1 pg/ml, *P* = 0.004). After 72 h, IL-10 was 46.0 ± 4.0 pg/ml in the supernatant of splenic lymphocytes from disease rats with IMRC co-culture, which was still higher than the other three groups (*P* < 0.01).

IL-10 expression was analyzed with different cell numbers of hECS-IMRCs at 48 h. When the ratio of splenocytes to IMRCs was 10:1 (2 × 10^5^:2 × 10^4^), IL-10 expression was 76.3 ± 6.5 pg/ml in the co-culture supernatant, which was higher than that in the supernatant with splenocytes and IMRC co-culture at 1:1 (2 × 10^5^:2 × 10^5^) (62.4 ± 3.7 pg/ml, *P* = 0.001), and that in splenocytes and IMRC co-culture at 100:1 (2 × 10^5^:2 × 10^3^) (24.8 ± 5.8 pg/ml, *P* < 0.001) (Fig. 7).

## Discussion

To our knowledge, this is the first study that MSCs were administrated on Heymann nephritis rats for the treatment of MN. We found that proteinuria was reduced remarkably to a level comparable to negative controls, in both low-dose and high-dose treatment groups. IgG and C3 deposit, glomerular basement membrane thickness and foot process effacement were alleviated, and nephrin expression was recovered in the kidneys. This efficacy was noted when IMRC treatment was administrated at week 3 after the rats presented with proteinuria. All these results demonstrate that IMRCs might have potential clinical values for the treatments of MN patients.

Clinical studies with primary MSCs derived from the umbilical cord, bone marrow or adipose tissue have been hampered by the lack of available donors, limited cell numbers from each donor, donor and tissue heterogeneity, inconsistent cell quality and the lack of standardized cell preparations. Moreover, primary MSCs show limited self-renewal capacities and finite lifespans. In the current study, we used IMRCs derived from self-renewing hESC cultures with serum-free reagents, which have unique merits to solve these problems [18]. IMRCs, while similar to primary UCMSCs, were superior in their long-term proliferative capacity, hyper-immunomodulatory and anti-fibrotic functions. The cell diameters of IMRCs were generally smaller than UCMSCs, suggesting that they pose lower risks for pulmonary embolism after injection. IMRCs did not engraft, nor transdifferentiate or initiate tumorigenesis, showing excellent potential for short- and long-term safety profiles by a range of in vitro and in vivo assays [20–23].

Primary MN is a kidney-specific autoimmune disease mediated by autoantibodies towards antigens on podocytes and the subsequent podocyte injuries resulting from complement activation [24]. After IMRC treatments, we found that the elevated level of C3 in the circulation of the rats with Heymann nephritis was significantly decreased. At the same time, the glomerular deposition of C3 and the resultant thickening of basement membrane and podocyte foot processes fusion were alleviated. These results provide evidence that IMRC treatment could inhibit the pathogenesis of MN and exert therapeutic effects. It is noted that the level of circulating C3 in rats of interventions was decreased to a similar level as that in negative controls, but not lower than that, which indicates that IMRC treatment does not lead to excessive consumption of complement, thus avoiding the side effects of increased infection risk associated with complement exhaustion therapies.

In the current study, we found that the proportions of CD4^+^CD25^+^ T lymphocytes in all CD4^+^ T cells were significantly increased in the detection of spleen and circulating T cell subsets after both doses of hESC-IMRC treatments. CD4^+^CD25^+^ T lymphocytes are the main cells in the regulatory T cells family. Its main functions include inhibiting autoimmune response, preventing injurious immunopathology, and maintaining immune balance [25, 26]. This finding suggested that IMRC infusion may play a therapeutic role by up-regulating or activating regulatory T cell subsets. It has been shown that MSCs can generate T regulatory cells in vitro, from activated human peripheral blood mononuclear cells, mouse splenocytes or isolated CD4^+^ T cells [27, 28]. Our experiments in vivo imply that this mechanism may also lead to a therapeutic effect of IMRCs in MN patients.

IL-10 plays an immunosuppressive role in various types of cells. After IMRC treatments, the circulating IL-10 level of rats with Heymann nephritis was significantly increased compared to those of disease controls and negative controls. At the same time, no difference was shown on IL-2 or INF-γ. It suggests that IMRCs may exert the immunosuppressive effect on MN by promoting a shift of T lymphocytes immune balance from Th1 to Th2, thereby reducing kidney injuries. This finding is consistent with the elevation of regulatory T cells after MSCs treatments. Treg inhibits the proliferation of juvenile and memory T cells mainly through the production of IL-10 [29].

We found that the circulating level of IL-17A and TNF-α were significantly increased in the rats with Heymann nephritis, and the treatments of IMRCs could reduce them. Bothe IL-17 and TNF-α are proinflammatory cytokines in the mechanism of MN. Previous studies [30] have found that the levels of IL-17 in the serum and kidney tissue of MN patients were increased remarkably, which effectively mediate the neutrophil mobilization and inflammatory response of tissues. The intrinsic cells in kidney, such as mesangial cells and endothelial cells, have the ability to produce and release TNF-α, which promotes the formation of NO and oxygen free radicals by stimulating iNOS, causing lipid peroxidation damage to cell membranes [31]. Peroxidative lipid metabolites alter the permeability of the glomerular basement membrane and lead to the formation of proteinuria [32]. Therefore, the reduction of inflammatory cytokines might be a mechanism of the therapeutic effects of IMRCs to MN.

The inhibitory effect of IMRCs on the proliferation of T lymphocytes is dose-dependent and time-dependent. In our co-culture assay, we designed the ratio of IMRCs and T lymphocytes at 1:10 and found that IL-10 level was increased after 24 h of co-culture, the highest at 48 h and decreased at 72 h. It may provide some references for the medication interval of IMRCs in potential clinical practice. Previous studies [33] have found that when the ratio of IMRCs to T lymphocytes is greater than 1:9, the inhibition effects occur. The inhibition effects disappear when the number of MSCs is less than 1% of T lymphocytes. Our results of the co-culture assay were consistent with that. We further assessed IL-10 level from the supernatant of IMRCs and lymphocytes with the ratio of 1:1, and found no increasement of IL-10 compared to that of IMRCs and lymphocytes 1:10. In the rats of Heymann nephritis, we also found similar therapeutic effects between two doses of IMRCs interventions. These results suggest to avoid an excessive amount of IMRCs in clinical usage for the lacking of further benefit but the risks of side effects.

After the IMRCs were administrated to the rats, the cells were rapidly taken up by the lungs. As time passes, the infused cells were taken up by the kidneys and spleen, and finally eliminated by the liver. In the kidneys, IMRCs were increased at 24 h and 48 h, and decreased after 72 h, which was probably due to the high blood flow rate in the kidneys. These results imply that the mechanism of IMRCs therapeutic effect may be through the immune modulation effects rather than cell proliferation in situ.

## Conclusions

In summary, our findings showed that intravenously delivered hESC-IMRCs significantly alleviated proteinuria and kidney injuries of Heymann nephritis in vivo. The mechanism may be the immunosuppressive effects of IMRCs through the upregulation of regulatory T cells and IL-10 and the inhibition of IL-17 and TNF-α. These pre-clinical results indicate that IMRCs are worth careful consideration for human trials in the treatment of MN.

## Supplementary Information

**Additional file 1**. **Figure**. Gating strategies for T cell subsets in flow cytometry.

## Data Availability

The datasets used and/or analyzed during the current study are available from the corresponding author on reasonable request.

## Abbreviations

MN: Membranous nephropathy
hESC: Human embryonic stem cells
IMRCs: Immunity-and-matrix regulatory cells
hESC-IMRCs: Human embryonic stem cells-derived immunity-and-matrix regulatory cells
IMRCs: Immunity-and-matrix regulatory cells
IgG: Immunoglobulin G
GFP: Green fluorescent protein
C3: Complement 3
CD4: Cluster of differentiation 4
CD25: Cluster of differentiation 25
IL-2: Interleukin-2
IL-4: Interleukin-4
IL-6: Interleukin-6
IL-10: Interleukin-10
MSCs: Mesenchymal stem cells
UCMSCs: Umbilical cord-derived MSCs
Fx1A: Proximal tubular epithelial cell brush border extract
GBM: Glomerular basement membrane
