# Supplementary figures and images for: The therapeutic effects of human embryonic stem cells-derived immunity-and-matrix regulatory cells on membranous nephropathy
Source: Stem Cell Res Ther. 2022 Jun 7;13:240. doi: 10.1186/s13287-022-02917-w (PMC9172125; doi:10.1186/s13287-022-02917-w)

## 1、Lymphocytes、CD45

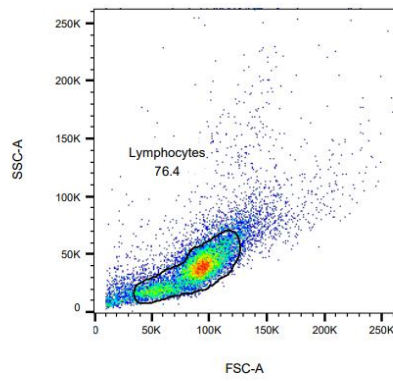

L-001.fcs  
Ungated  
11476

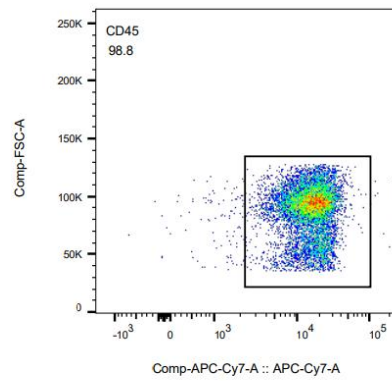

L-001.fcs  
Lymphocytes  
8771

## 2、CD3、CD4/CD8

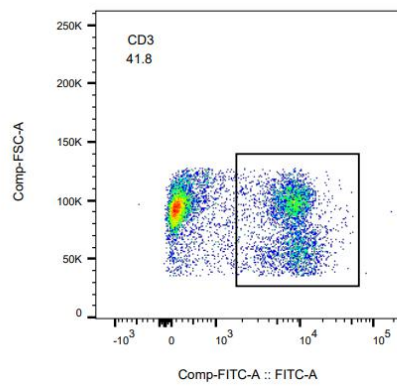

L-001.fcs  
CD45  
8663

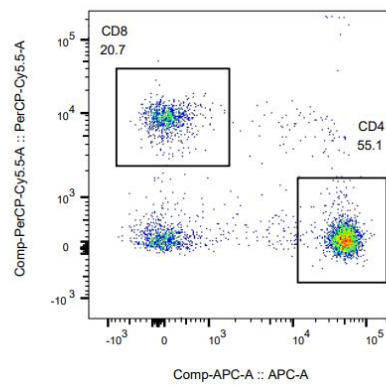

L-001.fcs  
CD3  
3624

## 3、CD4+CD25+

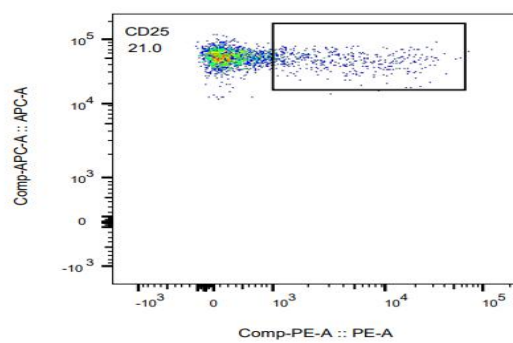

L-001.fcs  
CD4  
1998

Supplement: Supplementary file 1 — Additional file 1. Figure. Gating strategies for T cell subsets in flow cytometry. [file 13287_2022_2917_MOESM1_ESM.pdf]
